# Megakaryoblastic leukemia-1 is required for the development of bleomycin-induced pulmonary fibrosis

**DOI:** 10.1186/s12931-015-0206-6

**Published:** 2015-03-27

**Authors:** Ksenija Bernau, Caitlyn Ngam, Elizabeth E Torr, Benjamin Acton, Jacob Kach, Nickolai O Dulin, Nathan Sandbo

**Affiliations:** Department of Medicine, University of Wisconsin-Madison, Madison, WI USA; Department of Medicine, University of Chicago, Chicago, IL USA

**Keywords:** Pulmonary fibrosis, Myofibroblasts, MKL1, SRF, Bleomycin

## Abstract

**Background:**

Fibrosing disorders of the lung, such as idiopathic pulmonary fibrosis, are characterized by progressive extracellular matrix accumulation that is driven by myofibroblasts. The transcription factor megakaryoblastic leukemia-1 (MKL1) mediates myofibroblast differentiation in response to several profibrotic stimuli, but the role it plays in mediating pulmonary fibrosis has not been fully elucidated. In this study, we utilized mice that had a germline deletion of MKL1 (MKL1 (−,-)) to determine the role that MKL1 plays in the development of bleomycin-induced pulmonary fibrosis.

**Methods:**

Bleomycin or normal saline were intratracheally delivered to 9 to 12 week old female MKL1 (+,+) and MKL1 (−,-) mice. Mice were assessed for weight loss and survival to 28 days. Inflammatory responses were assessed through bronchoalveolar lavage at days 3 and 7 post-treatment. The development of pulmonary fibrosis was characterized using hydroxyproline assay and histological staining. MKL1 (+,+) and MKL1 (−,-) mouse lung fibroblasts were isolated to compare morphologic, gene expression and functional differences.

**Results:**

MKL1 (−,-) mice demonstrated increased survival, attenuated weight loss, and decreased collagen accumulation compared to wild-type animals 28-days after intratracheal instillation of bleomycin. Histological analysis demonstrated decreased trichrome, smooth muscle α-actin, and fibronectin staining in MKL1(−,-) mice compared to MKL1 (+,+) controls. Differential cell counts from bronchoalveolar lavage demonstrated that there was attenuated neutrophilia 3 days after bleomycin administration, but no difference at day 7. Isolated mouse lung fibroblasts from MKL1 (−,-) mice had decreased contractility and deposited less fibronectin matrix compared to wild-type controls, suggesting a defect in key remodeling functions.

**Conclusions:**

Altogether, these data demonstrate that MKL1 plays a significant role in mediating the fibrotic response to bleomycin injury. Loss of MKL1 attenuated early neutrophil influx, as well as myofibroblast-mediated remodeling. Targeting MKL1 activity may therefore be a useful strategy in treating pulmonary fibrosis.

## Background

Fibrosing disorders of the lung, such as idiopathic pulmonary fibrosis, are characterized by progressive extracellular matrix accumulation that is driven by myofibroblasts [1-4]. We have previously reported that myofibroblast differentiation requires signaling via the transcription factors megakaryoblastic leukemia-1 (MKL1) and serum response factor (SRF) [5-7]. MKL1 is regulated by the actin cytoskeleton [4], and activates the transcription factor SRF [8-10]. Additionally, MKL1 has been observed to be required for myofibroblast differentiation in response to mechanical signals, matrix stiffness, and profibrotic G-protein coupled receptor agonists [11-13]. Furthermore, our group has observed that signaling via protein kinase A (PKA) can protect against bleomycin-induced pulmonary fibrosis in mice, and that this protective effect is likely via inhibition of MKL1/SRF in myofibroblasts [14,15]. In this study, we examined the role of MKL1 in the development of pulmonary fibrosis using mice with a germline deficiency of MKL1 [16] in the bleomycin model of disease. We report that MKL1-deficient mice have decreased collagen accumulation and increased survival compared to wild-type animals. These effects are due, in part, to modulation of critical myofibroblast remodeling functions, such as contractility and fibronectin matrix formation.

## Methods

### Animals

Wild-type (MKL1 (+,+)) and knock out (MKL1 (−,-)) C57BL/6 mice [16] were kindly provided by Dr. Eric Olson (University of Texas-Southwestern). Mice were housed under controlled illumination and temperature with unlimited access to water and rodent laboratory chow. All animal studies were done following the appropriate University of Wisconsin-Madison Institutional Care and Use Committee and the National Institutes of Health guidelines using 9 to 13 week old female mice. Over the course of the experiments, the animals were closely monitored for overall health and activity, and their weight was taken daily.

### Bleomycin-induced pulmonary fibrosis

9 to 13 week old MKL1 (+,+) and MKL1 (−,-) female mice were anesthetized with ketamine (100 mg/kg) and xylazine (15 mg/kg) prior to delivering a single dose of intratracheal bleomycin (2 unit/kg, Sigma-Aldrich Co., St. Louis MO) dissolved in 50 μL of 0.9% NaCl Irrigation (Baxter, Madison, WI) via endotracheal intubation. Control animals were given a single intratracheal dose of 50 μL of 0.9% NaCl solution. Bronchoalveolar lavage (BAL) was performed on mice 3 or 7 days after bleomycin treatment, while the lungs of mice euthanized 28 days after treatment were used for the hydroxyproline assay (left lung) and histology (right lung). The mice were anesthetized using ketamine (150 mg/kg) and xylazine (22.5 mg/kg) and exsanguinated via the carotid artery prior to removing the lungs or BAL. Log-rank test was performed to analyze the differences in survival between bleomycin-treated MKL1 (−,-) and MKL1 (+,+) mouse groups.

### Hydroxyproline assay

Hydroxyproline assay was performed as described before [15,14]. Briefly, left lungs were isolated and homogenized in 0.5 M glacial acetic acid, followed by drying overnight at 60°C. Dried, whole lung homogenates then were weighed and hydrolyzed in 6 N HCl for 24 hours at 110°C. Triplicate-samples were incubated with citrate-acetate buffer and chloramine T at room temperature (RT) for 20 min prior to adding Ehrlich’s solution and incubating at 65°C for 1 hour. Sample absorbance was measured at 550 nm once the samples had cooled to RT. Using a standard curve generated from trans-4-Hydroxy-L-proline (Sigma-Aldrich), sample hydroxyproline content was determined. One-way ANOVA was performed to compare the collagen accumulation in the lungs of bleomycin-treated MKL1 (−,-) and MKL1 (+,+) mice.

### Histology

Right lungs were inflated using formaldehyde and fixed for 24–48 hours before being moved to 70% ethanol. The lungs were sectioned and subjected to Masson’s trichrome staining and immunostaining for smooth muscle α-actin (α-SMA) or fibronectin. Secondary antibodies conjugated to either HRP (immunohistochemical images) or Alexafluor-488 (for quantitation of α-SMA) were utilized. The modified Ashcroft scale was used to quantify fibrosis, as previously described [17]. Quantitation of α-SMA immunostaining was performed by taking 10 images at 20X magnification per sample, using identical exposure times and thresholds on an Olympus 1X71 fluorescent microscope and Q imaging Retiga 2000R camera. ImageJ was used to measure integrated density of the fluorescent signal [18]. Quantitation of fibronectin histology was performed by taking a 2X magnification image of each sample, using identical exposure times and thresholds on an Olympus SZH10 microscope and Olympus DP70 camera. ImageJ Immunohistochemistry Toolbox plugin was utilized to isolate the brown stain [19]. The resulting image was inverted, the lung was outlined and integrated density measured. Integrated density measurement was divided by the total area of the lung in order to account for differences in lung sizes.

### Bronchoalveolar lavage

3 or 7 days following intratracheal bleomycin or 0.9% NaCl treatment, BAL was performed. The mice were anesthetized and exsanguinated as described above. The trachea was then exposed and a catheter was used to intubate the mice. The lungs were lavaged 5 times using 1 ml of 0.9% NaCl. The BAL fluid was collected, centrifuged at 400 *g* for 10 min, the total cells in the pellet were counted and cytospins containing 7.5 x 10^5^ cells/slide prepared. Cytospins were stained using Wright Giemsa stain (Fisher Scientific, Kalamazoo, MI) and then counted noting the ratio of neutrophils, macrophages, lymphocytes and eosinophils out of 500 total cells. One-way ANOVA was performed to compare the number of each cell type between treatment groups.

### Isolation of mouse lung fibroblasts

Mouse lung fibroblasts were derived as previously described [5] and in accordance with established methodology [20]. Briefly, following extraction, the lung was immediately placed in cell maintenance medium: Dulbecco’s Modified Eagle Medium (DMEM) + L-glutamine (Corning Inc., Corning, NY), fetal bovine serum (FBS, 10%, Thermo Scientific, Waltham, MA), penicillin/streptomycin/amphotericin (PSA, 1%, Mediatech, Herndon, VA), Ciprofloxacin (1%, Corning Inc.) and L-glutamine (1%, Thermo Scientific). After several rinses, the lung was minced, washed in media and plated to attach. The media was replaced twice weekly until 80-90% fibroblast confluence was reached at which point the cells were trypsinized and passaged or utilized for studies. Negative cell staining for E-cadherin confirmed no contamination with epithelial cells. For experiments, cells were grown in 6-well plates at a density of 2 x 10^5^ cells/well for 24 hours, placed in starvation medium (maintenance medium with 10% FBS replaced by 0.1% bovine serum albumin (BSA)) for 24 hours. For indicated experiments, cells were treated with 1 ng/ml Transforming Growth Factor-β (TGF-β) (EMD Biosciences, Gibbstown, NJ) for the indicated times prior to being subjected to gel contraction assay or deoxycholate extraction.

### Immunofluorescent staining

Mouse lung fibroblasts (MLF) were washed with tris buffered saline (TBS), fixed for 30 min at RT using 4% paraformaldehyde in TBS, permeabilized for 5 min at RT using 0.2% Triton X-100 in TBS and blocked for 1 hour at RT in 10% Normal Goat Serum (NGS, Jackson Laboratory, Bar Harbor, ME), 1% BSA in TBS. The cells were incubated overnight at 4°C with the relevant primary antibody, washed with TBS and incubated for 75 min at 37°C with appropriate secondary antibody conjugated to fluorescein isothiocyanate (FITC, Pierce, Thermo Fisher, Rockford, IL). They were then washed and if required incubated with rhodamine phalloidin (Invitrogen, Carlsbad, CA) for 30 min at RT. Cells were counterstained with DAPI in TBS (0.42 μg/ml, Sigma-Aldrich) for 10 min at RT, washed and mounted using Vectashield mounting medium (Vector Labs, Burlingame, CA). Olympus 1X71 fluorescent microscope and Q imaging Retiga 2000R camera were used to obtain immunofluorescent images. To quantify focal adhesion length, 20 images at 60x magnification were taken per cell type and the length of vinculin stained focal adhesions was measured using ImageJ.

### Western blot

Cells were lysed in radioimmunoprecipitation assay (RIPA) buffer containing 25 mM HEPES (pH 7.5), 150 mM NaCl, 1% Triton-X 100, 0.1% SDS, 2 mM EDTA, 2 mM EGTA, 10% glycerol, 1 mM NaF, 200 μM Na-orthovanadate, 1 mM Na-phyrophosphate, 1 mM β-glycerol phosphate and protease inhibitor cocktail (Thermo-Scientific) for 10 min on ice. Next, cells were scraped, sonicated for 5 sec and centrifuged at 21.1 *g* for 10 min at 4°C. The supernatant was then mixed with Laemmli buffer and boiled for 5 min. The samples were electrophoresed on polyacrylamide gels at 150 V for 1 hour and transferred at 100 V for 1 hour. Western blot was run with desired primary antibodies and corresponding HRP-conjugated secondary antibodies. The blots were developed by an enhanced chemiluminescence (ECL) reaction (Pierce). GE LAS4000 chemiluminescence imager was used to obtain images below the saturation threshold. Densitometry of selected blots was performed using ImageGuage software (GE).

### Gel contraction assay

MLF were trypsinized and seeded in a 4 mg/mL rat-tail collagen solution (BD Biosciences, San Jose, CA) at a density of 3 x 10^5^ per mL in a 12-well plate. Gels were allowed to solidify, then released from the plate and allowed to contract in serum-free medium. Reduction of gel diameter was calculated in each condition using ImageJ.

### Deoxycholate (DOC) extraction

To determine the amount of fibronectin that is fully incorporated into the extracellular matrix by MLF, we assessed the amount of fibronectin that was resistant to deoxycholate extraction, as per established methodology [21]*.* Briefly, MLF (1 x 10^5^ per ml) were plated in 10 cm dishes for 24 hours in cell maintenance medium. Cells were then starved for 24 hours in starvation medium and treated with TGF-β (1 ng/mL) for 24 hours. Monolayers were washed and scraped into 2% DOC in TBS, 2 mM EDTA, 2 mM PMSF and protease inhibitor cocktail (Thermo-Scientific). Extracts were centrifuged at 21.1 *g* for 15 min at 4°C. DOC-soluble material was removed, and SDS-PAGE sample buffer (0.5 M Tris pH 6.8, 2% SDS, 10% glycerol) was added with and without the addition of β-mercaptoethanol and 10 mM DTT to reflect reduced or unreduced conditions. DOC-insoluble material was resuspended in solubilization buffer (1% SDS in TBS, 2 mM EDTA, 2 mM PMSF and protease inhibitors) and treated with sample buffer as described above. All samples were boiled for 5 minutes prior to being subjected to polyacrylamide gel electrophoresis on 8% gels and Western blotting.

### Reverse transcription quantitative real time PCR

Real time PCR was carried out as previously described [7]. Briefly, left lungs were pulverized over dry ice and 100 mg of tissue lysed in 1 ml RNA STAT-60 (AMS Biotechnology) to extract total RNA. 1 μg of total RNA was used as a template for random-primed reverse transcription using an iScript cDNA synthesis kit (Bio-Rad). Real time PCR analysis was performed using iTaq SYBR Green supermix with ROX (Bio-Rad) in an ABI 7500 multi-color real time PCR detection system (Applied Biosystems). PCR primers for MKL2 (also known as MRTFB) were CCCCAGCAGTTTGTTGTTCAGCACTCTT (forward) and GATGCTGGCTGTCACTGGTTTCATCTTG (reverse), for α-SMA were AAACAGGAATACGACGAAG (forward) and CAGGAATGATTTGGAAAGGA (reverse), for FN were AGACCATACCTGCCGAATGTAG (forward) and GAGAGCTTCCTGTCCTGTAGAG (reverse), and for Collagen 1α1 were ATGTTCAGCTTTGTGGACCT (forward) and CAGCTGACTTCAGGGATGT (reverse).

### Antibodies

Rabbit monoclonal antibody against α-SMA (for histological staining) was from Abcam (Cambridge, UK). Polyclonal rabbit antibody against fibronectin was a generous gift from Dr. Deane Mosher at the University of Wisconsin-Madison. Mouse monoclonal antibodies against α-tubulin, α-SMA and vinculin were from Sigma-Aldrich. Rabbit polyclonal antibody against total fibronectin was from Abcam. Mouse monoclonal antibody against GAPDH was from Santa Cruz Biotech (Santa Cruz, CA). Rabbit polyclonal antibody against total MKL1 was from Bethyl (Montgomery, TX). Mouse monoclonal antibody against E-Cadherin was from BD Biosciences (San Jose, CA).

### Statistics

One-way ANOVA was utilized to compare differences between group means for continuous variables. For survival data, we utilized the log-rank test.

## Results

### MKL1 is essential for the development of bleomycin-induced pulmonary fibrosis

**Figure 1:**
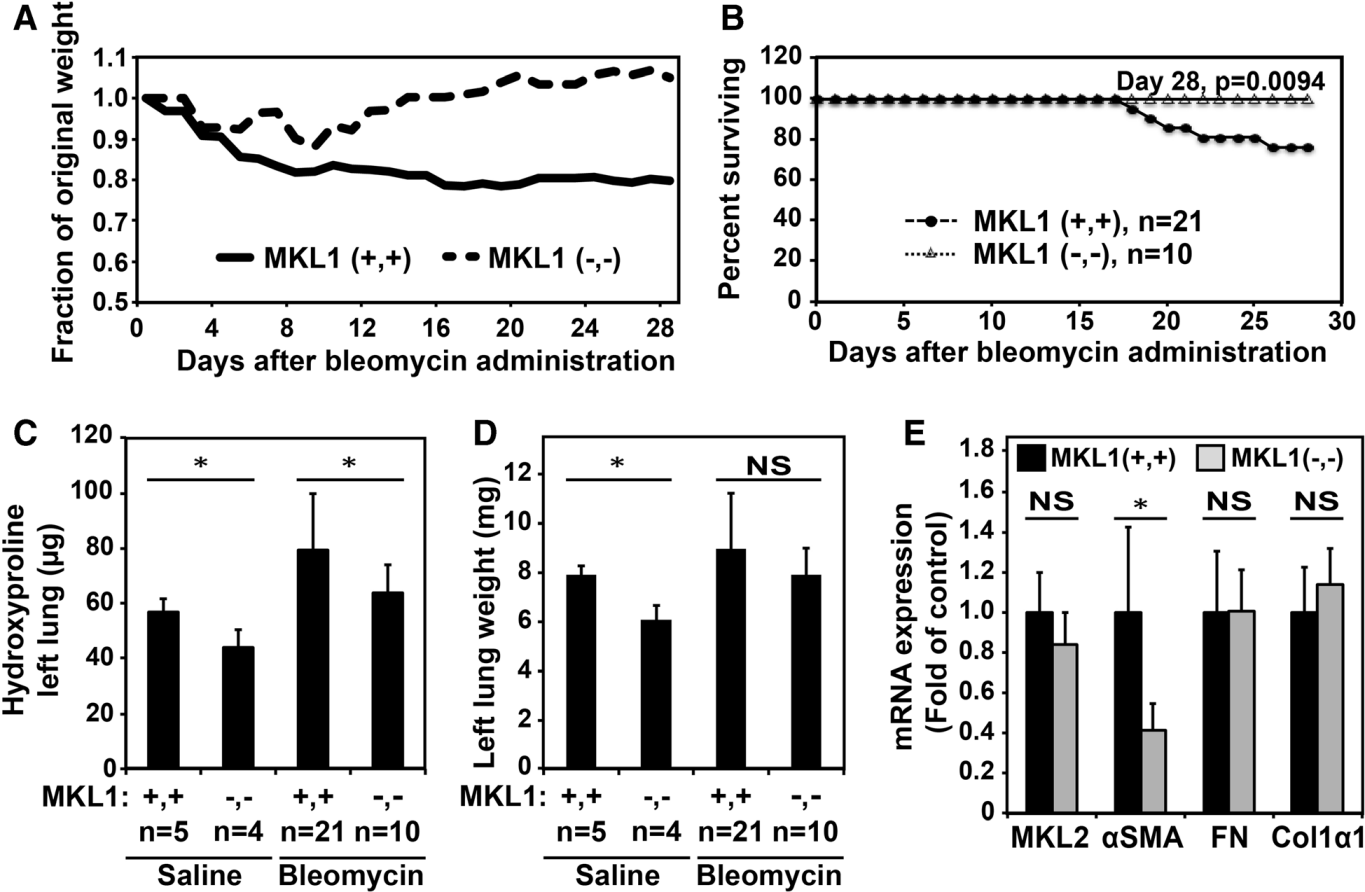
**MKL1 (−,-) mice demonstrate increased survival and decreased collagen accumulation following experimentally-induced fibrosis compared to MKL1 (+,+) controls. A**. Comparison of fraction of original weight over time (up to day 28) for MKL1 (+,+) and MKL1 (−,-) mice. **B**. Percent survival of MKL1 (+,+) and MKL1 (−,-) animals over time (up to day 28). Log-rank test (*p* < 0.05) was used to assess statistical significance. **C**. Quantitation of collagen accumulation in the left lung of MKL1 (+,+) and MKL1 (−,-) animals 28 days following saline or bleomycin treatment using hydroxyproline assay. **D**. Left lung dry weights of MKL1 (+,+) and MKL1 (−,-) animals treated with either normal saline or bleomycin. **E**. mRNA expression of MKL2, α-SMA, fibronectin and collagen1α1 in MKL1 (+,+) and MKL1 (−,-) untreated mouse lungs. One-way ANOVA (*p* < 0.05) was used to test for statistical significance.

We first explored the effects that MKL1 germline deletion has on the bleomycin-induced model of pulmonary fibrosis. Since MKL1/SRF are known mediators of myofibroblast differentiation [5-7], we hypothesized that loss of MKL1 would attenuate pulmonary fibrosis. Following treatment with bleomycin, MKL1 (+,+) and MKL1 (−,-) mice’ weights were monitored daily. We found that while the average MKL1 (+,+) animal weight decreased to approximately 80% of their initial weight, MKL1 (−,-) mice had similar initial weight loss, but then began to regain weight from the second week after bleomycin administration, correlating with the typical onset of the fibrotic response (Figure 1A). Furthermore, there was a significant difference in animal survival between the two groups, with less than 80% of MKL1 (+,+) animals surviving 28-days post bleomycin treatment in contrast to 100% survival of MKL1 (−,-) mice (Figure 1B). We then assessed total fibrosis at 28 days using the hydroxyproline assay to estimate collagen accumulation. Bleomycin-treated MKL1-deficient mice had significantly less collagen accumulation compared to wild-type controls (Figure 1C), while overall dry lung weights were similar between the groups (Figure 1D). We noted slightly lower levels of hydroxyproline content in MKL1 (+,+) versus MKL1 (−,-) mice not treated with bleomycin, but this was likely due to slightly smaller lungs in the knockout group (Figures 1C, D, bar 2). To determine whether there were potential basal differences in extracellular matrix gene expression between MKL1 (+,+) mice and MKL1 (−,-) mice, we assessed mRNA levels of fibronectin and collagen 1α1 in whole lungs of uninjured animals. As shown in Figure 1E, there were no significant differences in the basal expression of these genes between uninjured MKL1 (+,+) or MKL1 (−,-) lungs. In contrast, the expression of smooth muscle α-actin (α-SMA), a known target of MKL1, was significantly attenuated in uninjured MKL1 (−,-) mice. Notably, the expression of MKL2, which shares some signaling overlap with MKL1, was not significantly changed in MKL1 (−,-) mice, compared to wild type controls. These data demonstrate that MKL1 plays an important role in mediating the fibrotic response to bleomycin, while being dispensable for the expression of fibronectin and collagen 1α1 (but not of α-SMA) in uninjured lungs.

**Figure 2:**
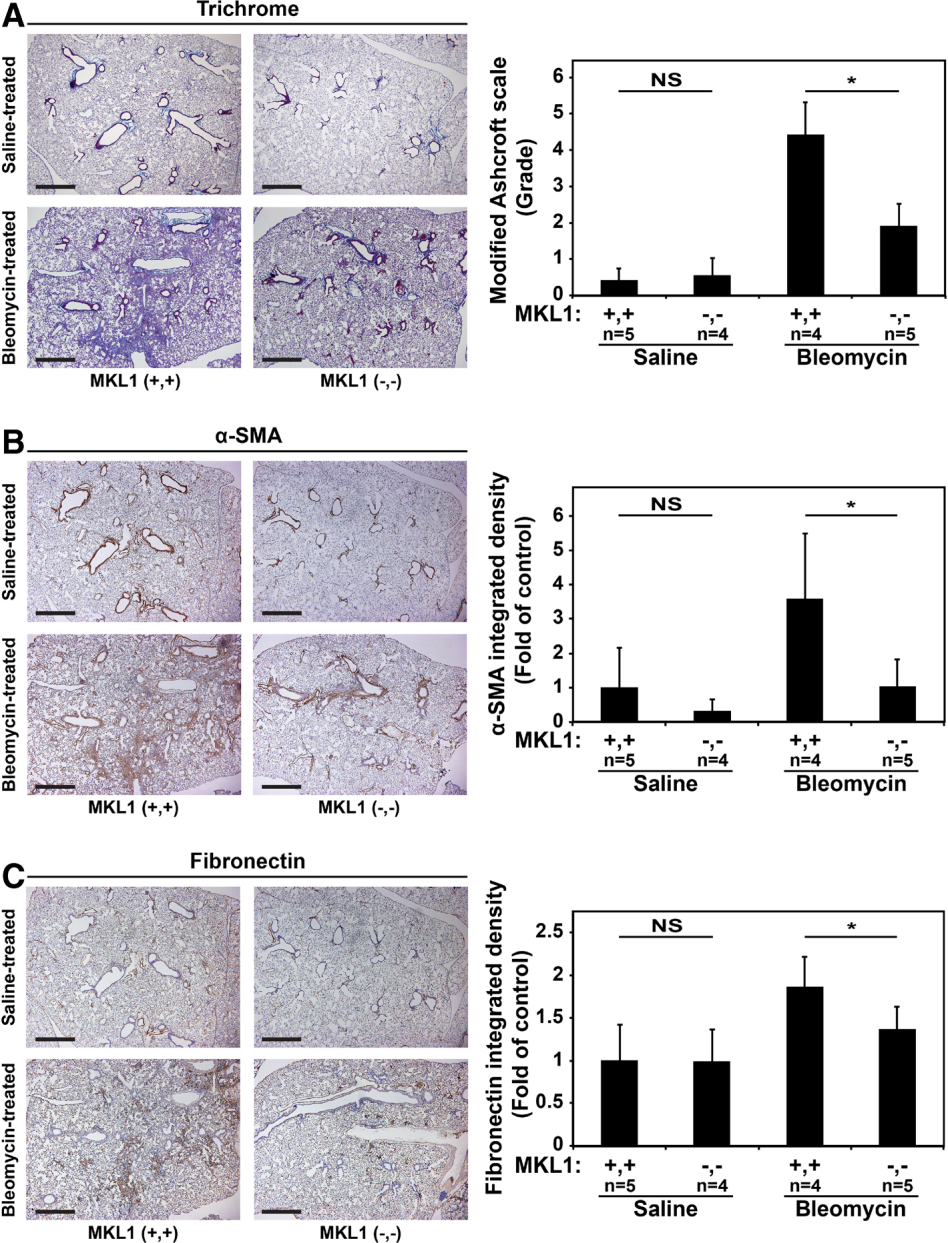
**MKL1 (−,-) mouse lungs following experimentally-induced fibrosis have decreased collagen, α-SMA and fibronectin accumulation compared to MKL1 (+,+) controls. A**. Trichrome staining of MKL1 (+,+) and MKL1 (−,-) mouse lungs following bleomycin or saline treatment denoting areas of collagen accumulation (blue) with the corresponding modified Ashcroft score for each condition. **B**. Immunohistochemical staining for α-SMA (brown) and **C**. fibronectin (brown) including their respective quantitation. One-way ANOVA (*p* < 0.05) was used for statistical significance.

Trichrome staining of mouse lungs at 28 days was consistent with the observed decrease in bleomycin-induced collagen accumulation seen in MKL1 (−,-) mice (Figure 2A). Fibrosis scoring of these slides, using modified Ashcroft scores [17], demonstrated a reduction in the fibrotic response to bleomycin in MKL1 (−,-) mice (Figure 2A, right panel). To assess the expansion of myofibroblasts in the lungs during bleomycin fibrosis, we utilized α-SMA staining. The lungs from bleomycin-treated MKL1 (+,+) mice had significantly more α-SMA staining compared to MKL1 (−,-) mice 28 days post bleomycin injury (Figure 2B). Fibroblast-mediated deposition of a fibronectin matrix is required for the formation of a collagen matrix [22,23]. Thus, to assess for *de novo* matrix accumulation, we stained for cellular fibronectin, which demonstrated decreased staining in bleomycin-treated MKL1 (−,-) mice compared to the wild-type controls (Figure 2C). Together, these results further indicate that MKL1 attenuates fibrosis following bleomycin treatment, possibly via inhibition of myofibroblast expansion and fibronectin matrix formation.

### MKL1 mediates neutrophilic inflammation and tissue injury in response to bleomycin

**Figure 3:**
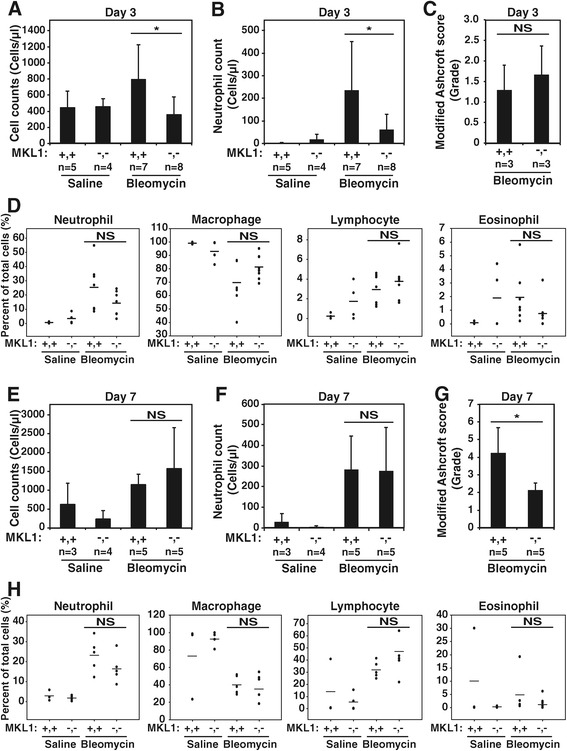
**The early inflammatory response to bleomycin does not differ between MKL1 (+,+) and MKL1 (−,-) mice. A**. Total BAL cell counts obtained 3 days following intratracheal saline or bleomycin treatment in MKL1 (+,+) and MKL1 (−,-) mice. **B**. Absolute neutrophil counts from the day 3 BALs obtained in Figure 3 **A**. **C**. Modified Ashcroft scores from trichrome-stained MKL1 (+,+) and MKL1 (−,-) mouse lungs done 3 days after bleomycin treatment. **D**. BAL differential cell counts of the same day 3 groups as in Figure 3 **A**, depicting the percentage of neutrophils, macrophages, lymphocytes and eosinophils counted following saline and bleomycin treatment in MKL1 (+,+) and MKL1 (−,-) mice. **E**. Total BAL cell counts obtained 7 days following intratracheal saline or bleomycin treatment in MKL1 (+,+) and MKL1 (−,-) mice. **F**. Absolute neutrophil counts from the day 7 BALs obtained in Figure 3 **E**. **G**. Modified Ashcroft scores from trichrome-stained MKL1 (+,+) and MKL1 (−,-) mouse lungs done 7 days after bleomycin treatment. **H**. BAL differential cell counts of the same day 7 groups as in Figure 3 **E**, depicting the percent of neutrophils, macrophages, lymphocytes and eosinophils counted following saline and bleomycin treatment in MKL1 (+,+) and MKL1 (−,-) mice. One-way ANOVA (*p* < 0.05) was performed to test for statistical significance.

Considering that the response to intratracheal bleomycin delivery is initially characterized by injury followed by neutrophilic inflammation [24-27], we sought to determine whether the early response to bleomycin was modified by MKL1. To evaluate this possibility, BAL was performed at 3 and 7 days following bleomycin or saline treatment. Interestingly, overall BAL cell counts were attenuated in bleomycin-treated MKL1 (−,-) mice compared to MKL1 (+,+) controls at day 3 (Figure 3A). Bleomycin-induced neutrophilia was evident, as expected, in wild-type animals [25,24], however, there appeared to be attenuated neutrophilia in the BAL of bleomycin-treated MKL1 (−,-) mice at day 3 (Figure 3B). Most of this difference was related to the drop in total cell count in MKL1 (−,-) mice, as differential counts of BAL cells did not reveal significant differences in the percentage of neutrophils, macrophages, lymphocytes or eosinophils between bleomycin-treated MKL1 (−,-) and MKL1 (+,+) mice (Figure 3D). To determine if changes in neutrophilia correlated with tissue injury, we assessed histologic changes via Ashcroft score. As shown in Figure 3C, there were no significant differences between MKL1 (+,+) and MKL1 (−,-) mice at day 3. By day 7, total lavage cell counts had risen further in bleomycin-treated animals and there was no difference in total cell counts between MKL1 (+,+) mice and MKL1 (−,-) mice (Figure 3E). We also did not observe a significant difference in neutrophilic inflammation by absolute neutrophil count or by percentage of BAL cells (Figure 3F and H). Likewise, the amount of lymphocytic inflammation was unaltered (Figure 3H). However, Aschroft scoring of bleomycin-injured lungs at day 7 demonstrated attenuation in tissue injury and fibrosis in MKL1 (−,-) mice compared to wild-type controls (Figure 3G). Taken together, these data suggest that MKL1 may impact the early neutrophilic response to bleomycin. By day 7, these early differences in BAL neutrophilia have resolved, but MKL1 (−,-) mice display evidence of attenuated tissue injury and fibrosis in response to bleomycin.

### MKL1 is required for contractile functions in fibroblasts

**Figure 4:**
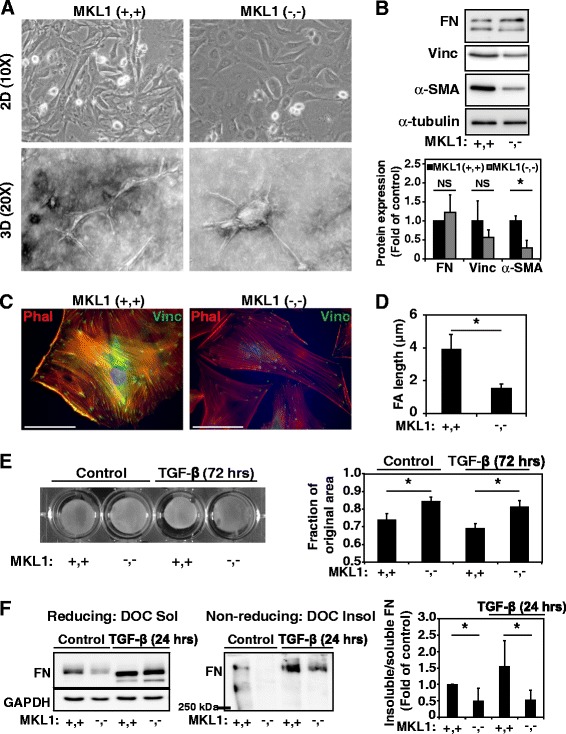
**MKL1 is required for contractile functions of the myofibroblast. A**. Phase contrast microscopy of representative MKL1 (+,+) and MKL1 (−,-) primary mouse lung fibroblasts (MLF) grown on 2D plates (top row) and 3D in collagen (bottom row) to show morphological differences. **B**. MKL1 (+,+) and MKL1 (−,-) primary MLF lysates were analyzed by Western blot with antibodies against fibronectin (FN), vinculin, α-SMA and α-tubulin. Densitometry of n = 3 experiments (bottom panel). **C**. Merged images of 2D primary MLF subjected to immunofluorescent staining with phalloidin rhodamine (red) and or primary antibodies directed against vinculin and FITC-conjugated secondary antibodies (green). **D**. Cells stained by indirect immunofluorescence against vinculin were analyzed for focal adhesion size by quantitation of vinculin plaque length using ImageJ (as described in methods). **E**. MKL1 (+,+) and MKL1 (−,-) primary MLF were treated with 1 ng/ml TGF-β (72 hrs) or vehicle control prior to plating into 3D collagen gels for gel contraction assessment over 2 days. Gel areas were quantified from the digital images using ImageJ (right panel). **F**. MKL1 (+,+) and MKL1 (−,-) MLF were plated, serum-starved and stimulated with 1 ng/ml TGF-β or vehicle control for 24 hours. Cells were subjected to deoxycholate (DOC) extraction, with DOC-soluble and DOC-insoluble lysates subjected to gel electrophoresis under reducing (first panel) and non-reducing (second panel) conditions followed by Western blotting for total fibronectin. Fibronectin bands from both soluble and insoluble lysates were quantified by densitometry and values expressed as the ratio of insoluble to soluble fibronectin (third panel). One-way ANOVA (*p* < 0.05) was performed to test for statistical significance.

The formation of a myofibroblast population during bleomycin pulmonary fibrosis is due to expansion and activation of resident lung fibroblasts [28-30]. Given the observed decrease in myofibroblasts seen in Figure 2, we sought to investigate the effects that MKL1 knockout has on primary mouse lung fibroblasts (MLF) *ex vivo*. Upon culturing in either a 2D or 3D system, MKL1 (−,-) MLF do not attain the typical spindle appearance that characterizes fibroblasts (Figure 4A). Rather the cells have a rounded, “epithelioid” morphology. Furthermore, we observed that MLF from MKL1 (−,-) mice exhibited decreased expression of the contractile gene α-SMA compared to MKL1 (+,+) MLF (Figure 4B), while fibronectin expression (two bands in Figure 4B representing two splice isoforms) appeared to be unaffected. Immunofluorescent co-staining of the actin cytoskeleton with phalloidin (red) and vinculin (green) revealed a decreased number and complexity of actin stress fibers in MKL1 (−,-) MLF as compared to MKL1 (+,+) controls (Figure 4C, rhodamine phalloidin stain, right image). Likewise, vinculin staining demonstrated smaller, more discrete focal adhesions (FA) by MKL1 (−,-) MLF in comparison to MKL1 (+,+) (Figure 4C, green stain, right image). This was confirmed through FA length analysis (vinculin staining) using ImageJ, showing that MKL1 (−,-) MLF indeed have shorter adhesions compared to MKL1 (+,+) MLF (Figure 4D). Functionally, MKL1 (−,-) cells demonstrated a defect in contraction of collagen gels, under basal conditions or after treatment with the profibrotic cytokine TGF-β for 72 hours (Figure 4E). Gel contraction quantification revealed a significant difference between MKL1 (+,+) and MKL1 (−,-) MLF under both control and TGF-β-stimulated conditions (Figure 4E, right panel). This effect is likely due to the loss of α-SMA expression (as in Figure 4B), which is critical for contractile force generation [31].

We then sought to determine whether key matrix remodeling functions of the fibroblast are affected by MKL1. Assembly of a mature, deoxycholate-insoluble fibronectin matrix is a key step in the formation of mature extracellular matrix [32]. TGF-β induces the formation of a deoxycholate (DOC)-insoluble fibronectin matrix in wild-type cells (Figure 4F, middle panel), as well as an increase in unincorporated, soluble fibronectin (Figure 4F, left panel). Under treatment with TGF-β, MKL1 (−,-) fibroblasts still manifest an increase in available soluble fibronectin (Figure 4F, left panel, lane 4, top band), but there is a loss of incorporation of fibronectin into a multimeric, DOC-insoluble matrix (Figure 4F, middle panel, lane 4). Quantitation of the ratio of DOC-insoluble matrix to DOC soluble matrix for MKL1 (+,+) and MKL1 (−,-) cells (Figure 4F, right panel), shows a reduction in the formation of a mature, DOC-insoluble fibronectin matrix by MKL1 (−,-) MLF under basal and TGF-β-stimulated conditions. Thus, despite an ability to express fibronectin, MKL1 (−,-) fibroblasts appear to have a defect in the ability to form a mature extracellular fibronectin matrix. This is also likely due, in part, to the loss of α-SMA expression which is required for the proper assembly and deposition of fibronectin [33,34]. Overall, these results show that MKL1 is required for maintaining both a contractile phenotype and key contraction-dependent remodeling functions in fibroblasts.

## Discussion

The present study demonstrates a key role of MKL1 in the development of pulmonary fibrosis in the bleomycin model of disease. Our results are consistent with the findings from other groups supporting a role for MKL1 in cardiac fibrosis [35] and in collagen accumulation in the lung in response to bleomycin [36]; and we further elaborate on the mechanism of the pro-fibrotic role of MKL1. Given the role that MKL1 plays in mediating myofibroblast differentiation, our primary hypothesis was that loss of MKL1 would impair myofibroblast-driven responses to bleomycin-injury. However, MKL1/SRF has also been shown to control neutrophil migration [37], lymphocyte maturation [38], and megakaryocyte maturation [39-41], each of which could modify the response to bleomycin. In fact, we did observe that MKL1 may facilitate the early neutrophilic response to bleomycin, as MKL1 (−,-) mice had less BAL neutrophilia at day 3 post-bleomycin administration. However, differences in BAL neutrophilia had resolved by day 7. In contrast to the observed changes in early BAL neutrophilia, we did not observe significant differences in other inflammatory cell counts at either day 3 or 7. Interestingly, while we did not see meaningful differences in tissue injury at day 3, we did observe significant changes in fibrosis score occurring by day 7 post-bleomycin administration. This suggests that the attenuated early neutrophilic response may translate to decreased tissue injury at early timepoints in MKL1 (−,-) mice. Despite these observations, it is important to note that the initial physiologic response to bleomycin during the early inflammatory phase is similar between the two groups, as MKL1 (−,-) mice experienced weight loss that initially paralleled their wild-type littermate controls. Weights began to diverge between the two populations around the second week, which coincides with the onset and peak of the fibrotic phase [27,26]. This suggests that modification of the fibrotic response is playing an important role in the protection seen in MKL1 (−,-) mice. Supporting this concept, markers of fibrosis, such as hydroxyproline content, myofibroblast expansion, and fibronectin matrix formation were all attenuated in the MKL1-deficient mice.

Our *ex vivo* experiments suggest that the attenuation in fibrosis seen in MKL1 (−,-) mice appears to be directly related to alterations in contractile gene expression, contractile force generation and the formation of a mature fibronectin matrix in MKL1 (−,-) fibroblasts. Taken together, these data suggest that the primary defect in MKL1 (−,-) animals is that of impaired fibroblast remodeling functions leading to a decreased amount of fibrosis. Notably, MKL1 (−,-) mice have also been observed to have defects in lactation, due to loss or dysfunction of myoepithelial cells, contractile cells that are similar to myofibroblasts [16,42]. The observed loss of the contractile gene α-SMA in MKL1 (−,-) fibroblasts may be critical, as loss of α-SMA also results in failure of lactation [43,44] and we have recently reported that myofibroblast-mediated fibronectin matrix assembly requires expression of α-SMA [33]. Our work lends further support to the importance of contractile gene expression by myofibroblasts in mediating their function. An additional component to the attenuation of fibrosis seen in MKL1 (−,-) mice could be the loss of anti-apoptotic signaling via the SRF-dependent gene, BCL2 [45] or attenuated expression of collagen isoforms [35,46]. For example, potentiation of myofibroblast apoptosis via attenuation of BCL2 expression has been postulated in mediating the protective effect of the ROCK inhibitor fasudil in the bleomycin model [36]. These mechanisms would be expected to add to the defects in fibroblast remodeling functions that we observed in this model.

In our studies, the protective response of MKL1 germ-line deletion appears to be partial. We hypothesize that this could be explained by compensatory signaling via MKL2, a homologue of MKL1 which can also mediate activation of SRF-dependent genes and myofibroblast differentiation [47,48,13]. MKL2 compensatory signaling in MKL1-null animals has been previously reported in differentiation of other cell types, supporting this hypothesis [39]. Importantly, we did not see any upregulation of MKL2 levels in MKL1 (−,-) mice (Figure 1E), but redundant signaling through MKL2 could still partially compensate for loss of MKL1. Unfortunately, germ-line deletion of MKL2 cannot be explored in the bleomycin model due to embryonic lethality [49,50].

Inhibition of MKL1-dependent signaling is potentially attractive given its role as an integrator of several profibrotic inputs [4], and the potential for preservation of TGF-β signaling that may serve a homeostatic role [51,52]. Nuclear localization of MKL1 is controlled by actin dynamics, under the control of Rho-kinase (ROCK) [10] and ROCK is required for bleomycin-induced pulmonary fibrosis [53,36]. Thus, the protective effect of MKL1 germline deletion we have observed is consistent with a vital role for the ROCK/MKL1 signaling axis in the fibrotic response. However, ROCK signaling is pleotropic and can also be involved in regulating Smad [54,55] and MAP kinase signaling [56,57], suggesting that these two molecules do not have completely overlapping effects. Furthermore, although the protective effect of MKL1 deletion is likely due, in large part, to loss of SRF-dependent gene expression during fibrosis, we cannot completely exclude contributions from Smad-dependent genes, given the reported interaction of Smads and MKL1 [58]. Thus, future approaches that utilize tissue-restricted deletion of MKL1 or SRF in adult animals may better clarify the best approach to disrupting this signaling axis.

## Conclusions

Altogether, our study indicates that MKL1/SRF may play a significant role in neutrophil influx, myofibroblast differentiation, and lung fibrosis in response to bleomycin, as MKL1-deficient mice demonstrate decreased evidence of collagen accumulation and increased survival compared to wild-type controls. Our *ex vivo* analysis confirms that attenuated lung fibrosis is due, in part, to MKL1’s critical role in myofibroblast remodeling functions, such as fibronectin incorporation and contractile force generation. In light of this, targeting MKL1/SRF may be a useful strategy for disrupting myofibroblast function during pulmonary fibrosis.

## Abbreviations

HEPES: 4-(2-hydroxyethyl)-1-piperazineethanesulfonic acid
DAPI: 4’-6-diamidino-2-phenylindole
ANOVA: Analysis of variance
BCL2: B-cell lymphoma 2
BSA: Bovine serum albumin
BAL: Bronchoalveolar lavage
DOC: Deoxycholate
DTT: Dithiothreitol
DMEM: Dulbecco’s Modified Eagle Medium
ECL: Enhanced chemiluminscence
EDTA: Ethylene diamine tetraacetic acid
EGTA: Ethylene glycol tetraacetic acid
FBS: Fetal bovine serum
FN: Fibronectin
FITC: Fluorescein isothiocyanate
FA: Focal adhesions
GAPDH: Glyceraldehyde 3-phosphate dehydrogenase
HRP: Horseradish peroxidase
MKL1: Megakaryoblastic leukemia-1
MKL1 (−,-): Megakaryoblastic leukemia-1 knock-out mice
MKL1 (+,+): Megakaryoblastic leukemia-1 wild type mice
MLF: Mouse lung fibroblasts
NGS: Normal goat serum
PSA: Penicillin/streptomycin/amphotericin
PMSF: Phenylmethanesulfonyl fluoride
PCR: Polymerase chain reaction
PKA: Protein kinase A
RIPA: Radioimmunoprecipitation assay
ROCK: Rho-associated protein kinase
RT: Room temperature
SRF: Serum response factor
α-SMA: Smooth muscle α-actin
SDS-PAGE: Sodium dodecyl sulfate polyacrylamide gel electrophoresis
TGF-β: Transforming growth factor-βTransforming growth factor-β
TBS: Tris buffered saline
